# *Annona muricata* Leaf as an Anti-Cryptosporidial Agent: An In Silico Molecular Docking Analysis and In Vivo Studies

**DOI:** 10.3390/ph16060878

**Published:** 2023-06-14

**Authors:** Eman S. El-Wakil, Hagar F. Abdelmaksoud, Majed H. Wakid, Muslimah N. Alsulami, Olfat Hammam, Haleema H. Albohiri, Marwa M. I. Ghallab

**Affiliations:** 1 Department of Parasitology, Theodor Bilharz Research Institute, Imbaba 12411, Egypt; pearlhfn@yahoo.com; 2 Department of Medical Laboratory Sciences, Faculty of Applied Medical Sciences, King Abdulaziz University, Jeddah 21589, Saudi Arabia; mwakid@kau.edu.sa; 3 Special Infectious Agents Unit, King Fahd Medical Research Center, King Abdulaziz University, Jeddah 21589, Saudi Arabia; 4 Department of Biology, College of Science, University of Jeddah, Jeddah 21589, Saudi Arabia; mnal-sulami@uj.edu.sa (M.N.A.); hhalbahiri@uj.edu.sa (H.H.A.); 5 Department of Pathology, Theodor Bilharz Research Institute, Imbaba 12411, Egypt; totoali1@hotmail.com; 6 Department of Medical Parasitology, Faculty of Medicine, Kafrelsheikh University, Kafrelsheikh 33516, Egypt; marwaghalab@gmail.com

**Keywords:** *Cryptosporidium*, immunocompromised mice, *Annona muricata*, nitazoxanide immunohistochemistry

## Abstract

Cryptosporidiosis is a serious parasitic diarrheal disease linked to the occurrence of colorectal cancer in immunocompromised patients. The FDA-approved drug nitazoxanide (NTZ) achieved a temporary effect, and relapses occur. *Annona muricata* leaf is widely used in traditional medicine to treat a wide range of disorders, including antiparasitic and anticancer effects. So, this study aimed to investigate *Annona muricata* leaf antiparasitic and anticancer properties compared to NTZ in *Cryptosporidium parvum* (*C. parvum*) acutely and chronically infected immunosuppressed mice. A molecular docking analysis was performed to evaluate the effectiveness of some biologically active compounds that represented the pharmacological properties of *Annona muricata* leaf-rich extract toward *C. parvum* lactate dehydrogenase compared to NTZ. For the in vivo study, eighty immunosuppressed albino mice were classified into four groups as follows: group I: infected and treated with *A. muricata*; group II: infected and treated with nitazoxanide; group III: infected and received no treatment; and group IV: were neither infected nor treated. Furthermore, half of the mice in groups I and II received the drugs on the 10th day post-infection (dpi), and the other half received treatment on the 90th day post-infection. Parasitological, histopathological, and immunohistochemical evaluations were performed. The docking analysis showed that the lowest estimated free energy of binding of annonacin, casuarine, L-epigallocatechin, P-coumaric acid, and ellagic acid toward *C. parvum* LDH, were −6.11, −6.32, −7.51, −7.81, and −9.64 kcal/mol, respectively, while NTZ was −7.03 kcal/mol. Parasitological examination displayed a significantly high difference in *C. parvum* oocyst mean counts in groups I and II compared to group III (*p*-value < 0.001), with group I demonstrating the highest efficacy. The analyses of histopathological and immunohistochemical results revealed that group I showed restoration of the normal villous pattern without evidence of dysplasia or malignancy. *A. muricata* leaf has proved to be a reliable agent for *Cryptosporidium* treatment. This paper argues for its promising use as an antiparasitic agent and for the prevention of neoplastic sequels of *Cryptosporidium* infection.

## 1. Introduction

*Cryptosporidium parvum* (*C. parvum*) is a widely distributed protozoan parasite that can infect the gastrointestinal epithelium of both humans and other mammals [1]. Waterborne outbreaks are the main route of infection in addition to the fecal-oral route. Together with the documented low infectious dose and the resistance of the oocysts to chlorine disinfection, these factors contribute to its waterborne transmission, making it a major health problem [2].

Cryptosporidiosis presentation differs according to the host’s immune status. In immunocompetent hosts, it is usually asymptomatic, or it may result in self-limited diarrhea that resolves spontaneously without medications [3]. In immunocompromised hosts (e.g., AIDS and children under one year), *Cryptosporidium* could be responsible for severe watery diarrhea with growth retardation or life-threatening consequences [4].

In general, there is a link between chronic inflammation and cancer. Particularly in the immunocompromised host, *C. parvum* produces a number of pathological alterations in gastrointestinal epithelial cells and is associated with an increased risk of acquiring colorectal cancer [5]. However, the concept of considering chronic *Cryptosporidium* infection as the catalyst for tumor formation is supported by a small amount of data, and those findings were primarily derived from animal research [6,7].

Nitazoxanide is the present standard cryptosporidiosis treatment. It accelerates recovery in immunocompetent individuals but unfortunately has a poor effect on children and is even ineffective in patients with AIDS [8]. Therefore, optimal therapy for cryptosporidiosis, especially in immunocompromised hosts, is urgently needed.

Many parasite diseases, including cryptosporidiosis, have been treated using medicinal herbs [9]. They include *Asafoetida* [10], *Ficus carica* [11], *Olea europaea* [11], *Verbena Officinalis* ([12], *Egyptian propolis* [13], and *Herniaria hemistemon* [1].

*Annona muricata* (*A. muricata*) is the heart-shaped, edible fruit of the family *Annonaceae* found in both tropical and subtropical regions. Its different parts, seeds, roots, and leaves have a significant role in herbal medicine [14]. *A. muricata* extracts have been worthy and promising in antiparasitic and anticancer medicine. Their effects include cytotoxicity, necrosis, proliferation inhibition, and apoptosis induction on various cancer cell lines, including colorectal ones [15,16]. The key biomolecules mastering the *A. muricata* bioactive conditions are mainly acetogenins and alkaloids [17]. Moreover, *A. muricata* had been tested for its antiprotozoal properties, including *Toxoplasma gondii*, *Leishmania* spp., *Trypanosoma cruzi*, and *Plasmodium falciparum* [16]. The anti-helminthic effect of *A. muricata* was also documented on *Trichinella spiralis* [18].

As dysplastic gastrointestinal tract changes could be a consequence of both infection and inflammation, specific markers are essential. A significant number of colorectal tumors, particularly those with high degree dysplasia, have significant aberrant expression of retinoblastoma protein, cyclins D1, D2, and cyclin-dependent kinase inhibitor p16 [19]. Among these markers is cyclin D1, a crucial protein that regulates the cell cycle, and in the case of its upexpression, the normal cell cycle control will be interrupted, thus participating in the pathogenesis of cancer. Since cyclin D1 level evaluation is reproducible, it can be considered an efficient prognostic marker [20].

In biomedical research, molecular modeling has become crucial for reducing lab effort and aiding the identification of the most likely molecular targets and/or signaling pathways. Since the molecular docking technique may significantly increase productivity and save research costs, it has gained popularity recently. Additionally, predicting the binding affinity and examining the interaction mode have become essential tools in computer-assisted drug design [21].

In the present work, we have attempted to assess *A. muricata* leaf extract’s antiparasitic and anticancerous effects compared to NTZ in *C. parvum* acutely and chronically infected immunosuppressed mice.

## 2. Results

### 2.1. Docking Simulation Analysis Results

**Figure 1:**
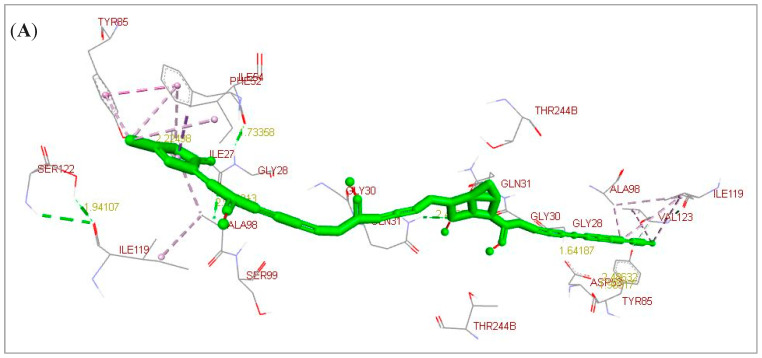

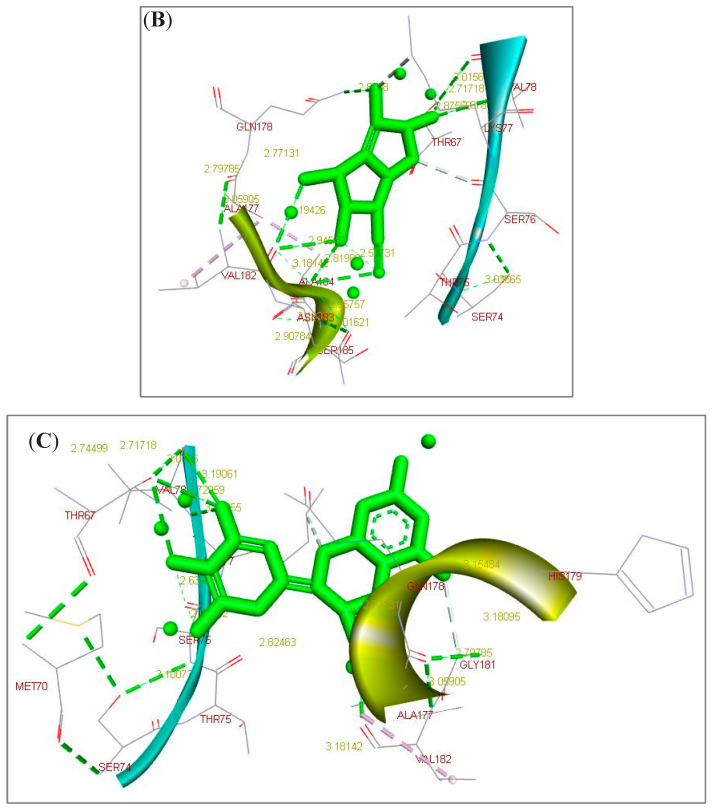
3D docked structures toward *Cryptosporidium parvum* lowa II lactate dehydrogenase (LDH) by using BIOVIA Drug Discovery Studio Visualizer software. (**A**) Annonacin (acetogenin). (**B**) Casuarine (alkaloid). (**C**) L-Epigallocatechin (phenolics/flavonoid). Dotted light green lines represented the ligand-binding site atoms, dotted dark green and grey lines showed the inter- and intramolecular and ligand- amino acid residue H-bonds, and dotted light purple lines demonstrated the electrostatic, hydrophobic, and other interactions.

**Figure 2:**
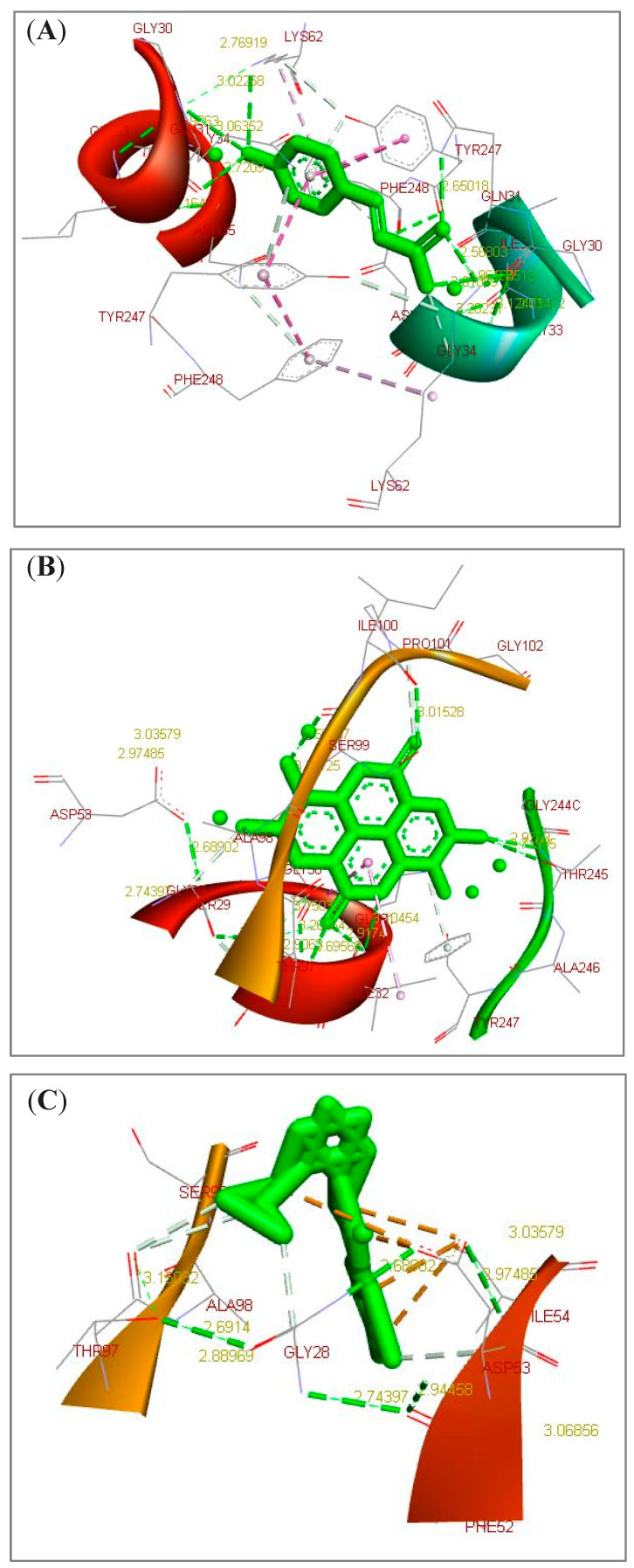
3D docked structures toward *Cryptosporidium parvum* lowa II lactate dehydrogenase (LDH) by using BIOVIA Drug Discovery Studio Visualizer software. (**A**) P-Coumaric acid (phenolics/phenolic acid). (**B**) Ellagic acid (phenolics/Ellagic tannins). (**C**) Nitazoxanide (NTZ, standard reference drug). Dotted light green lines represented the ligand-binding site atoms, dotted dark green and grey lines showed the inter- and intramolecular and ligand- amino acid residue H-bonds, and dotted light purple lines demonstrated the electrostatic, hydrophobic, and other interactions.

*Annona muricata* leaves include phenolics, alkaloids, and acetogenins that potentially represent several bioactive compounds with many medicinal therapeutic properties. As reported in Table 1, *A. muricata* leaves clearly include elevated levels of annonacin, casuarine, L-epigallocatechin, P-coumaric acid, and ellagic acid. They have a potent estimated free energy of binding (−6.11, −6.32, −7.51, −7.81, and −9.64 kcal/mol, respectively) and great estimated inhibition constant (Ki) values (33.48, 23.25, 3.12, 1.88 µM, and 85.42 nM, respectively) toward *C. parvum* LDH compared to the nitazoxanide (NTZ) standard drug (−7.03 kcal/mol and 7.06 µM, respectively). According to the scoring functions, the strength of the docked ligands is potentially correlated to the intermolecular interactions between the binding partners (ligands and their target) as H-bonds and electrostatic and hydrophobic interactions. As an acetogenin, annonacin formed interactions with LDH by using H-bonds (GLY28.B and GLN31.B) and electrostatic and hydrophobic interactions (GLY28, ILE54, TYR85, ALA98, ILE119, and VAL123) (Figure 1A). As an alkaloid, casuarine interacted with LDH by using H-bonds (THR67.B, SER74.B, VAL78.B, GLN178.A, VAL182.A, ASN183.A, and ALA184.A) (Figure 1B). As a phenolic compound, L-epigallocatechin interacted with LDH by using H-bonds (THR67.B, SER76.B, VAL78.B, GLN178.A, and VAL182.A) and electrostatic and hydrophobic interactions (GLY181) (Figure 1C). As a phenolic compound, P-coumaric acid formed interactions with LDH by using H-bonds (GLY30.A, GLN31.B, LYS62.A, and LYS62.B) and electrostatic and hydrophobic interactions (ASN35, LYS62, TYR247, and PHE248) (Figure 2A). As a phenolic compound, ellagic acid correlated with LDH by using H-bonds (ILE32.A, GLY33.A, THR97.A, SER99.A, ILE100.A, and THR245.A) and electrostatic and hydrophobic interactions (GLN31 and PRO101) (Figure 2B). As a standard reference drug, NTZ interacted with LDH by using H-bonds (ASP53.A) and electrostatic and hydrophobic interactions (GLY28, SER29, ASP53, ALA55, THR97, ALA98, and SER99) (Figure 2C and Table 1). As additional findings, UCSF Chimera visualization software (Version 1.16) was used to confirm these results (Supplementary Materials, Supplementary Figures S1 and S2).

### 2.2. Oocyst Shedding

Regarding the acute infection, treatment with *A. muricata* leaf significantly reduced the oocyst count compared to the nitazoxanide-treated infected group. The oocyst count decreased on the 12th day PI compared to the 10th day PI (group I: 83 vs. 16.3). This reduction continued to the end of the experiment in a significant way (group I: 16.3, 14, and 12.3 on the 12th, 15th, and 20th day PI, respectively). The nitazoxanide-treated group II achieved a 64, 67, and 70% reduction in comparison to their corresponding infected untreated group III (Table 2).

In the same way, treatment with *A. muricata* leaf during the chronic infection significantly reduced the oocyst count by 78, 80, and 83% on the 92nd, 95th, and 100th day PI, respectively. In the nitazoxanide-treated group II, the percent reduction was 57, 59, and 62% on the 92nd, 95th, and 100th day PI, respectively (Table 3).

In both Table 1 and Table 2, *Cryptosporidium* oocysts count per gram stool was represented as (mean ± SE) × 10^3^. The reduction was displayed as a percentage depending on the following equation: %: percent of reduction = [(mean count in the control infected group − mean count in the study group)/mean count in the control infected group] × 100.

In each row, the mean values marked with the same superscript small letter were similar, whereas those with different ones were significantly different. In each column, the mean values marked with the same superscript capital letter were similar, whereas those with different ones significantly differed. PI: post-infection and PR: percent of reduction.

### 2.3. Histopathological Findings

**Figure 3:**
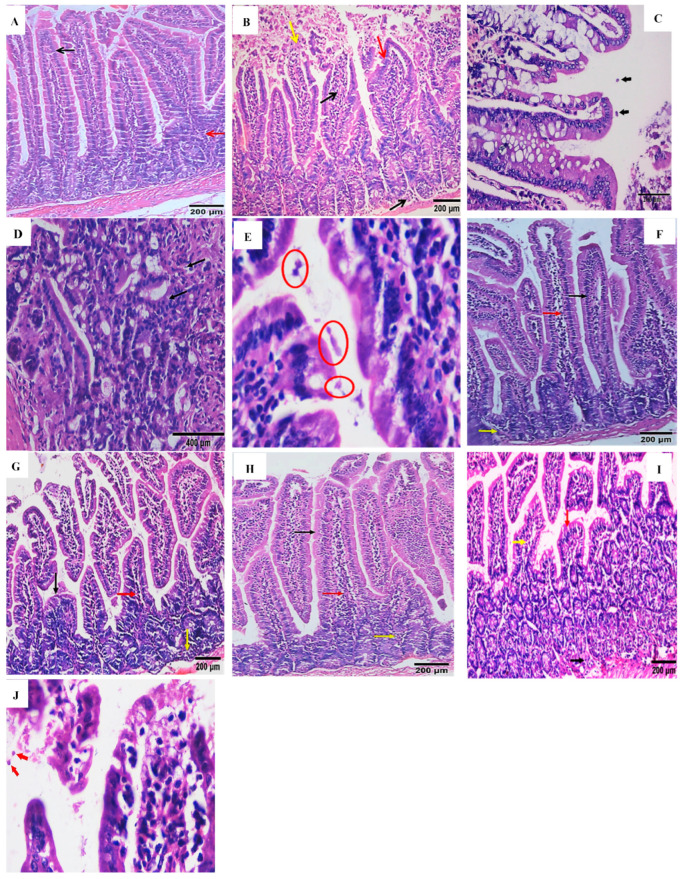
Sections of the small intestine of different study groups (H&E, ×200, unless mentioned otherwise). (**A**) GIV showed a normal structure of the mucosa (black arrow), and lamina propria (red arrow) with a normal small intestinal crypt villous ratio. (**B**) GIII on the 20th day post-infection showed moderate blunting and shortening of villous in the mucosa (red arrows), ulcerations (yellow arrow), non-specific inflammatory infiltration of the lamina propria, and villous cores (black arrow). (**C**) *Cryptosporidium* oocysts are either separate or adherent (black arrows) (H&E, ×400). (**D**,**E**) GIII on the 100th day showed intestinal mucosa with dysplastic gland changes in the form of enlarged nuclei and an increased nucleocytoplasmic ratio (black arrow), many *Cryptosporidium* oocysts along the surface epithelium (red circles), (H&E, ×1000). (**F**) GI on the 20th day showed an almost normal villous pattern (black arrow), mild non-specific inflammatory response noticed in villi (red arrow), and lamina propria (yellow arrow). (**G**) GII on the 20th day showed mild blunting and shortening of villous in the mucosa (black arrow), mild nonspecific inflammatory response noticed in villi (red arrow), and lamina propria (yellow arrow). (**H**) GI on the 100th day showed a near normal villous pattern (black arrow), moderate non-specific inflammatory response noticed in villi (red arrow), and lamina propria (yellow arrow). (**I**,**J**) GII on the 100th day displayed moderate blunting and shortening of villous in the mucosa (red arrows), moderate non-specific inflammatory infiltration of the lamina propria (black arrow), in villi (yellow arrow), and *Cryptosporidium* oocysts along the surface epithelium (red arrows) (H&E, ×1000).

Histopathological alternations in H&E-stained intestinal sections from various groups are shown in Figure 3A–J. Sections from the normal control group (GIV) showed normal architecture, healthy mucosa, and an average crypts/villous ratio. There was a moderate number of goblet cells with a well-defined brush border and lamina propria (Figure 3A).

Sections of the infected control (GIII) group, sacrificed on 20th day post-infection, displayed villous architecture loss, broadening, shortening, and even atrophy of the villi. Moreover, the lamina propria showed mucosal ulceration and non-specific inflammatory infiltration with inflammatory cells, mainly lymphocytes. Along the epithelial cells, *Cryptosporidium* oocysts appeared as purple-stained rounded to oval bodies, measuring 4–6 µm (Figure 3B,C). On the 100th day post-infection, the infected control group showed intestinal mucosa with dysplastic gland changes in the form of enlarged nuclei and increased nucleocytoplasmic ratios, and a mild lymphocytic inflammatory response was noticed in villi lamina propria with many *Cryptosporidium* oocysts along the surface epithelium (Figure 3D,E).

Regarding the acute infection, examined sections from the *A. muricata*-treated group (Figure 3F) and the nitazoxanide-treated group (Figure 3G) showed an obvious decrease in the intensity of the inflammatory cell infiltration, as well as a remarkable improvement of the other intestinal histopathological changes, with a return of the normal villous pattern.

There was a near-normal villous pattern in the chronic infection with moderate regenerative changes and a mild lymphocytic inflammatory response noticed in the villi and lamina propria, and there were no *Cryptosporidium* oocysts in the *A. muricata*-treated group (Figure 3H). In the nitazoxanide-treated group (Figure 3I), the intestinal mucosa had mild dysplastic changes in the form of hyperplasia of the villous epithelium, mildly enlarged nuclei, and a mild lymphocytic inflammatory response noticed in villi and lamina propria with scattered *Cryptosporidium* oocysts (Figure 3J).

### 2.4. Immunohistochemical Studies

**Figure 4:**
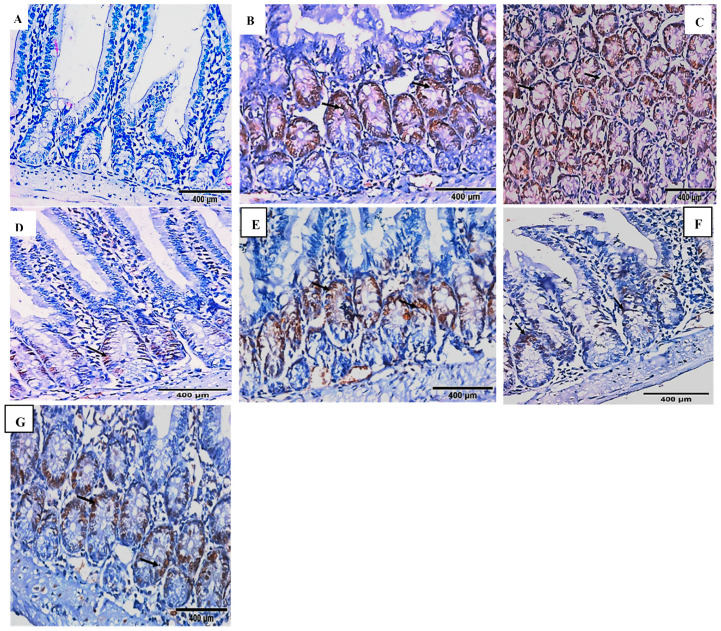
Nuclear expressions of cyclin D1 of different study groups (IHC, DAB, cyclin D1, ×400). (**A**) GIV showed a negative expression of cyclin D1. (**B**) GIII on the 20th day post-infection showed moderate positive nuclear expression of cyclin D1 in the crypts (black arrows). (**C**) GIII on the 100th day showed marked positive nuclear expression of cyclin D1 in all villi and crypts (black arrows). (**D**) GI on the 20th day showed a mild positive nuclear expression of cyclin D1 in the crypts (black arrow). (**E**) GII on the 20th day showed moderate positive nuclear expression of cyclin D1 in the crypts (black arrows). (**F**) GI on the 100th day showed a mild-moderate positive nuclear expression of cyclin D1 in the crypts (black arrows). (**G**) GII on the 100th day showed moderate positive nuclear expression of cyclin D1 in the crypts (black arrows).

Nuclear staining of the epithelial cells of the intestinal walls with cyclin D1 of different studied groups is shown in Figure 4A–G.

In the normal control group, there was a negative expression of cyclin D1 (Figure 4A). In the infected control group (GIII), on the 20th and 100th day post-infection, moderate and marked positive nuclear expression of cyclin D1 was detected, respectively (Figure 4B,C).

In the acute infection, sections examined from the *A. muricata* treated group (Figure 4D) showed mild expression of cyclin D1, while the nitazoxanide treated group (Figure 4E) showed a mild positive nuclear expression of cyclin D1.

In the chronic infection, sections examined from the *A. muricata* treated group (Figure 4F) showed a mild positive nuclear expression of cyclin D1, and the nitazoxanide-treated group (Figure 4G) showed a moderate positive nuclear expression of cyclin D1.

## 3. Discussion

*Cryptosporidium parvum* is considered one of the documented etiological protozoa for diarrheal disease that could be threatening, especially in immunocompromised hosts [22]. It was also linked with gastrointestinal malignancy [23]. Due to the reported antiparasitic effect of *A. muricata* and its high content of acetogenins [24], together with the anticancer properties of the *A. muricata* acetogenins and other metabolites, such as alkaloids, flavonoids, and others [25], this work is an attempt to evaluate the potential antiparasitic and anticancer properties of *A. muricata* leaf extract in immunocompromised mice.

According to the docking results, *A. muricata* leaves included several bioactive compounds that potentiated and confirmed its in vivo therapeutic properties toward *C. parvum* lactate dehydrogenase compared to NTZ. This was also supported by the in vivo study.

In our study, a highly statistically significant difference in the oocysts count was found among the nitazoxanide- and *A. muricata*-treated groups compared to the positive control groups (*p* < 0.001), with a reduction percentage of 70% and 86% in the nitazoxanide- and *A. muricata*-treated groups after 20 days post-infection, respectively. This result ensures that the *A. muricata* antiparasitic effect is superior to nitazoxanide. This effect is also explored in the group that remains infected for 100 days and is then treated with *A. muricata*, leading to a decrease in the oocysts counts, 83%, compared to the positive control group.

Sections of the small intestine of *Cryptosporidium*-infected animals that were then treated with *A. muricata* during the acute infection showed a normal villous pattern with a noticed mild lymphocytic inflammatory response in the villi and lamina propria. The immunohistochemistry analysis showed scattered positive cells in the intestinal crypt.

The antiparasitic effect of *A. muricata* was previously described. The extract of *A. muricata* and some of its isolated active metabolites are highly efficient against parasites incorporated in human diseases [24]. Ferreira et al. [24] tested the effectiveness of *A. muricata* extract against *Haemonchus contortus* eggs *(H. contortus)* in vitro. They concluded that the tested extracts are moderately effective against *H. contortus* eggs. There were previous investigations for the evaluation of the effect of the genus *Annona* and its extract against parasitic infections. Souza et al. [26] tested *A. squamosa* extract against cattle nematodes in vitro, and they reported inhibition of egg hatching (19.4%). Moreover, the same plant extract was tested in vivo with sheep by Vieira et al. [27]. They found a considerable decrease in the *H. contortus* egg counts (40%).

Previous research has demonstrated that *A. muricata* has an antiparasitic activity in vitro against *P. falciparum* [28], *T. cruzi*, and *Leishmania* spp. [14].

Somsak et al. [29] documented evidence of in vivo anti-malarial activity of *A. muricata*. This activity was attributed to the effective action of some of this plant’s bioactive compounds (diterpenoids, flavonoids, polyphenols, saponins, alkaloids, kaempferol, and acetogenin). Additionally, the antioxidant effect of A. *muricata* may also contribute to its anti-malarial activity [30]. Furthermore, it has been reported that phenolic compounds inhibit the activity of key enzymes for fatty acid biosynthesis [31]. Some of those mechanisms may be applied in the case of *C.parvum* infection, resulting in a reduction in oocyst counts.

Graviola offers a broad spectrum of safety. According to previous research, the oral median LD50 was calculated to be around 2500 mg/kg, and mice fed 4000 mg/kg did not die within the observation period of seven days [29]. 

The immunohistochemistry analysis revealed many positive cells lining the intestinal crypts. In agreement with our results, multiple previous studies reported histological changes in the intestinal epithelium attributed to the infection with *C. parvum* in animal models, such as distal small intestine crypt hyperplasia and villous atrophy [3,5,32].

The parasitic infestation-related changes exhibited at the region of the ileum in this study agree with the findings of others [3,5]. It has been documented that those ileal conditions, such as the biochemical environment and the availability of the needed receptors, favor *C. parvum* growth and development.

No evidence of carcinoma growth was found in this study. This is inconsistent with the study of Certad et al. [5], which described the occurrence of cryptosporidiosis-associated mucosal carcinoma lesions and parasite infection-related changes in the immunocompromised *C. parvum* infected mice [5]. This difference could be attributed to the fact that they used a more powerful method for immunosuppression than ours, as they relied on the severe combined immunodeficiency (SCID) mice model in addition to dexamethasone.

Verdon et al. [33] proposed that the higher the immunosuppression degree, the higher probability of neoplastic transformation. However, the study of Abdou et al. [3], like ours, did not show carcinoma development. They used dexamethasone only for immunosuppression.

Several mechanisms were proposed to explain the neoplastic effect of *C. parvum*. The chronic inflammatory process induced by the parasite leads to the subsequent reactive oxygen species (ROS) release and is involved in the occurrence of the mutation [34]. Additionally, there was a significant correlation between the *C. parvum* endogenous oocysts number and the development of high-grade intestinal dysplasia, demonstrating the impact of the parasite on carcinogenesis [6]. Another carcinogenic mechanism was reported by Certad et al. [5]. They described *C. parvum*-related angiogenesis, which is the ability of neovascularity creation. *C. parvum* infection was demonstrated to modulate the tumor suppressor genes’ expression and activity. The immunohistochemical analysis revealed abnormal signaling pathway components and p53. Additionally, adherents’ junction’s ultrastructure alterations occur in the ileocaecal epithelium of mice infected with *C. parvum*. Modulation of the host cytoskeleton network was also reported to be associated with *C. parvum*-induced carcinogenesis [7]. Regarding the immunohistochemical analysis, the dysplastic nuclei of dysplastic cells were strongly stained for cyclin D1. There was a high nuclear expression in the infected untreated group. This is consistent with the study by Abdou et al. [3], who documented an increase in cyclin D1 level in the dysplastic colonic epithelium of the infected control group, concluding that cyclin D1 is a good and useful marker for the detection of intestinal dysplasia.

## 4. Materials and Methods

### 4.1. Molecular Docking Simulation Analysis

Molecular docking is considered an important method that analyzes the conformation and orientation of ligands into the binding sites of their targets. Searching algorithms generate poses that are ranked according to their scoring functions [35,36]. To evaluate the effectiveness of some biologically active compounds from *A. muricata* leaf-rich extract, the molecular docking simulation analysis was carried out between the annonacin (MF: C_35_H_64_O_7_, CID:354398), casuarine (MF: C_8_H_15_NO_5_, CID:9859098), L-epigallocatechin (MF: C_15_H_14_O_7_, CID:72277), P-coumaric acid (P-hydroxy cinnamic acid, MF: C_9_H_8_O_3_, CID:637542), or ellagic acid (benzoaric acid, MF: C_14_H_6_O_8_, CID:5281855) and the crystal structure of *C. parvum* lactate dehydrogenase (LDH, PDB DOI: 4ND1) [37] compared to nitazoxanide (NTZ, MF: C_12_H_9_N_3_O_5_S, CID:41684) as a standard reference drug. The protein structure file (PDB file) was obtained from the RCSB-PDB database (http://www.rcsb.org/) (accessed on 6 March 2023). The structures of annonacin, casuarine, L-epigallocatechin, P-coumaric acid, ellagic acid, and NTZ were obtained from the PubChem database (https://pubchem.ncbi.nlm.nih.gov/) (accessed on 6 March 2023) and were then drawn by ACD/ChemSketch and converted to PDB files by using OpenBabel v2.3.2. Protein–energy minimization was carried out by using the Swiss-PDBViewer v4.1.0 program. Autodock 4.2.6, a molecular docking simulation tool, was utilized to explore the estimated ligand–protein free energy of the binding (ΔG, Kcal/mol). The grid box properties were 0.375 Å spacing, 13.531X-, 40.148Y-, and 78.91Z-center, and 140 was the number of points in the X-, Y-, and Z-dimensions. If the binding energy is <−5 kJ/mol, it represents that the target has a certain binding affinity toward the ligand [38,39,40,41]. For the docked ligands, the elevated negative values of the estimated free energy of the binding are positively correlated with their binding affinities and docking properties. After docking, the ligand (annonacin, casuarine, L-epigallocatechin, P-coumaric acid, ellagic acid, or NTZ) with the lowest binding energy toward *C. parvum* LDH was selected to visualize its docked form by using BIOVIA Drug Discovery Studio Visualizer (Version 21.1.0.20298) and UCSF Chimera (see Supplementary Materials) software(Version 1.16).

### 4.2. Experimental Animals

Eighty female, white Albino CDI strain mice (laboratory-bred, 4–6 weeks old, weighing 20–25 g each) were obtained from the biological unit of the Theodor Bilharz Research Institute (TBRI). This experiment was carried out at the TBRI animal house according to valid international guidelines.

### 4.3. Animal Groups

Mice were divided into four groups: 20 mice each. Group I was immunocompromised, infected, and treated with *A. muricata* leaf; group II was immunocompromised, infected, and treated with NTZ; group III was immunocompromised, infected, and untreated (control positive); and group IV was immunocompromised, non-infected, and non-treated (control negative).

Each group was subdivided into two subgroups (a and b); each consisting of ten animals to assess the effectiveness of the drugs given during the acute stage after ten days of infection (a) (10–15 days post-infection) and during the chronic stage after 90 days of infection (b) (90–95 days post-infection) separately.

### 4.4. Induction of Immunosuppression

The experimental mice were chemically immunosuppressed by the administration of dexamethasone sodium phosphate (Dexazone) at a dosage of 0.25 mg/g/day orally via an oesophageal tube for two weeks before the induction of infection; then, they continued to receive the same dose of dexamethasone throughout the experiment [42].

### 4.5. Induction of Cryptosporidium Infection

*Cryptosporidium parvum* oocysts were concentrated from the feces of infected calves after natural infection in our earlier research, which was confirmed genetically as *C. parvum* [43]. Oocysts were isolated and purified from stool samples according to [44], preserved in a 2.5% solution of potassium dichromate, and kept at 4 °C until required. To determine how much fluid volume was in the inoculum per mouse, the infective inoculum was prepared, and the number of infective oocysts in the concentrated stock inoculum was counted. Mice were infected orally with the oocysts via oral-gastric gavage. Each mouse received *Cryptosporidium* oocysts at a dose of approximately 10^4^ oocysts/mouse [45].

### 4.6. Drug Preparation and Regimens

Nitazoxanide (NTZ) (100 mg/5 mL suspension; Medizen Pharmaceutical Industries for Utopia Pharmaceuticals) was given at a dose of 100 mg/kg daily for five consecutive days [46].

*Annona muricata* leaf was obtained as a standardized 500 mg commercial vegetarian capsule. It was administrated for five consecutive days at a dose of 1 g/kg body weight dose [18,29]. Each capsule contains 1 g of *A. muricata* leaf, and due to its free radical scavenging properties, the manufacturer recommends its supportive beneficial effects on healthy cell growth and function. It was dissolved in distilled water and was given by gavage orally. Each mouse received the calculated dose in 0.2 mL.

The used drugs were administrated in two regimens as follows:

The first treatment regimen for acute infection began on the 10th day post-infection, and the mice were sacrificed on 20th day post-infection. The second treatment regimen for chronic infection began on the 90th day post-infection, and the mice were sacrificed on the 100th day post-infection.

### 4.7. Drugs Assessment

#### 4.7.1. Parasitological Examination

Following drug administration, fecal pellets from the infected mice were collected on the 10th, 12th, 15th, and 20th day post-infection for acute infection and on 90th, 92nd, 95th, and 100th day post-infection for chronic infection. The fecal pellets were examined parasitologically using cold Kinyoun’s acid-fast stain to count the number of *cryptosporidium* oocysts [47]. The fecal samples were collected from each infected mouse. The mean oocysts’ number for each mice group was calculated. One mg of the fecal pellet was weighed and preserved in 1 mL of 10% formalin. The fecal suspension was concentrated through centrifugation for 10 min at 500× *g*. Oocysts count in 1 mL of fecal sample was detected in 100 µL of dried and stained fecal sediment, and then examined under the oil immersion lens (×100). To determine the count of oocysts in one mL of fecal pellet, the average of 3 counts was calculated and multiplied by 10 [48]. The number of oocysts was expressed per gram of feces [6]. According to Hosking et al. [49], the following equation was used to calculate each drug’s efficacy as a percentage reduction:

PR (%) = ((mean value of the infected untreated group − mean value of infected treated group)*100)/(mean value of infected untreated group).

#### 4.7.2. Histopathological Examination

The subgroups (a) and (b) of mice were sacrificed on the 20th and 100th day post-infection, respectively, by light isoflurane inhalation anesthesia (Forane^®^, Baxter, Deerfield, MA, USA). The excised ileocecal segments were fixed in a 10% buffered formalin solution and were then embedded in paraffin wax blocks. After sectioning, staining using Hematoxylin and Eosin (H&E) was carried out according to Drury and Wallington [50].

#### 4.7.3. Immunohistochemical Examination

According to Hsu et al. [51], sections from the ileocecal region were immunohistochemically stained by cyclin D1 using rabbit monoclonal anti-human cyclin D1 (Cat# 54-0017, CA 94080) (Oncogene, San Francisco, CA, USA) in addition to a detection kit DAKO LSAB^®^ System HRP (DAB, DAKO, Glostrup, Denmark).

### 4.8. Statistical Analysis

Data were analyzed using Microsoft Excel 2016 and a statistical package for social science (IBM SPSS Statistics for Windows, version 26, IBM Corp., Armonk, NY, USA). Quantitative data were expressed as mean ± SD. ANOVA and Duncan’s multiple range test as a post hoc test were used to detect statistically significant differences among all the study groups. A *p*-value < 0.05 was significant and a *p*-value < 0.001 was highly significant.

## 5. Conclusions

*Cryptosporidium* is not only impacting health by its known implication in the resulting diarrheal disease, it also has a precancerous effect. *A. muricata* has proved to be an efficient agent for *Cryptosporidium* treatment. In addition to its documented anticancerous effect, this paper argues for its promising use as an antiparasitic agent with both infection eradication and the prevention of further neoplastic sequels. In addition, *A. muricata* decreases the expression of cyclin D1 in early and late infection and can be used as a prognostic marker in the case of colorectal dysplasia.

## Figures and Tables

**Table 1:** The in silico molecular docking characteristic properties of the interacted ligands were reported.

Lead/Ligand @	Binding Affinity #	Ki	Lead-Target Protein Binding and Interactions (Non-Covalent Intermolecular Interactions)
Annonacin	−6.11	33.48 *	H-bonds (GLY28.B and GLN31.B).
Casuarine	−6.32	23.25 *	Electrostatic and hydrophobic interactions (GLY28, ILE54, TYR85, ALA98, ILE119, and VAL123).
L-epigallocatechin	−7.51	3.12 *	H-bonds (THR67.B, SER74.B, VAL78.B, GLN178.A, VAL182.A, ASN183.A, and ALA184.A).
P-coumaric acid	−7.81	1.88 *	H-bonds (THR67.B, SER76.B, VAL78.B, GLN178.A, and VAL182.A).
Ellagic acid	−9.64	85.42 ^Δ^	Electrostatic and hydrophobic interactions (GLY181).
NTZ	−7.03	7.06 *	H-bonds (GLY30.A, GLN31.B, LYS62.A, and LYS62.B).

@, *C. parvum* lactate dehydrogenase LDH as a target protein; #, scoring function/binding affinity/estimated free energy of binding (kcal/mol); Ki, estimated inhibition constant; *, µM; ^Δ^, nM.

**Table 2:** *Cryptosporidium* oocysts count in different groups during the acute stage.

Groups	10th Day PI		12th Day PI		15th Day PI		20th Day PI	
	M ± SE × 10^3^	PR%	M ± SE × 10^3^	PR%	M ± SE × 10^3^	PR%	M ± SE × 10^3^	PR%
GI	83 ± 1.4 ^aA^	1%	16.3 ±. 8 ^bC^	81%	14 ± 1.6 ^cC^	84%	12.3 ±1.1 ^dC^	86%
GII	82.9 ± 1.3 ^aA^	1%	30.8 ± 1.3 ^bB^	64%	29.1 ± 1.1 ^cB^	67%	27 ± 1.9 ^dB^	70%
GIII	83.9 ±. 9 ^aA^	-	84.4 ± 1.9 ^aA^	-	87.5 ± 1 ^aA^	0	90 ± 2.2 ^aA^	-

**Table 3:** *Cryptosporidium* oocysts count in different groups during the chronic stage.

Groups	90th Day PI		93rd Day PI		95th Day PI		100th Day PI	
	M ± SE × 10^3^	PR%	M ± SE × 10^3^	PR%	M ± SE × 10^3^	PR%	M ± SE × 10^3^	PR%
GI	114.6 ± 3.4 ^aA^	0%	26.1 ± 1.8 ^bC^	78%	23.7 ± 1 ^cC^	80%	21.1 ± 1 ^dC^	83%
GII	115.1 ± 3.2 ^aA^	0%	50 ± 1.9 ^bB^	57%	48.9 ± 3.2 ^cB^	59%	46.1 ± 1.5 ^dB^	62%
GIII	115.3 ± 3.1 ^aA^	-	117.1 ± 2.5 ^aA^	-	118.6 ± 4.3 ^aA^	-	121.9 ± 4.2 ^aA^	-

## Data Availability

All data generated or analyzed during this study are included in this published article and its supplementary information files.

## Supplementary Materials

The following supporting information can be downloaded at: https://www.mdpi.com/article/10.3390/ph16060878/s1, Figure S1: 3D docked structures toward Cryptosporidium parvum lowa II lactate dehydrogenase (LDH) by using UCSF Chimera visualization software. A. Annonacin (acetogenin). B. Casuarine (alkaloid). C. L-Epigallocatechin (phenolics/flavonoid). Light blue lines represented the inter- and intramolecular H-bonds while orange lines demonstrated the relax H-bond constraints; Figure S2: 3D docked structures toward Cryptosporidium parvum lowa II lactate dehydrogenase (LDH) by using UCSF Chimera visualization software. A. P-Coumaric acid (phenolics/phenolic acid). B. Ellagic acid (phenolics/Ellagic tannins). C. Nitazoxanide (NTZ, standard reference drug). Light blue lines represented the inter- and intramolecular H-bonds while orange lines demonstrated the relax H-bond constraints.

## Abbreviations

ANOVA: analysis of variance; µL: microliter; µM: micrometer; *A. muricata*: *Annona muricata*; AIDS: acquired immune deficiency syndrome; *C. parvum*: *Cryptosporidium parvum*; dpi: day post-infection; FDA: Food and Drug Administration; g: gram; kcal/mol: kilocalorie per mole; H&E: Hematoxylin and Eosin; kg: kilogram; Ki: inhibition constant; LDH: lactate dehydrogenase; mg: milligram; mL: milliliter; nM: nanometer; NTZ: nitazoxanide; SE: standard error: TBRI; Theodor Bilharz Research Institute.
