# Supplementary material for: Annona muricata Leaf as an Anti-Cryptosporidial Agent: An In Silico Molecular Docking Analysis and In Vivo Studies
Source: Pharmaceuticals (Basel). 2023 Jun 14;16(6):878. doi: 10.3390/ph16060878 (PMC10303196; doi:10.3390/ph16060878)
Supplement: Supplementary file 1 [file pharmaceuticals-16-00878-s001.zip › pharmaceuticals-2438160-supplementary.pdf]

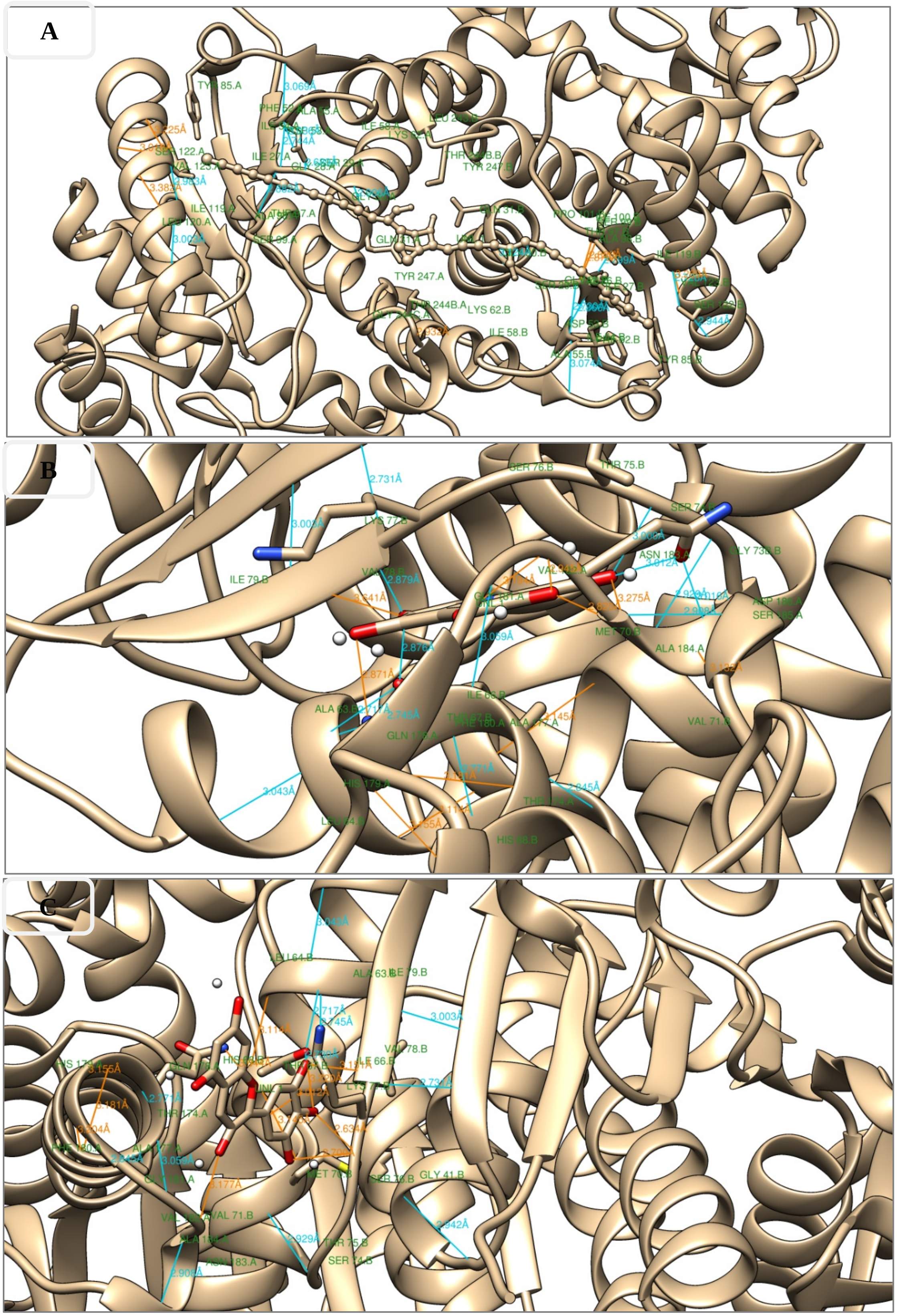
A
B
C

Supplementary Figure S1. 3D docked structures toward *Cryptosporidium parvum* lowa II lactate dehydrogenase (LDH) by using UCSF Chimera visualization software. **A.** Annonacin (acetogenin). **B.** Casuarine (alkaloid). **C.** L-Epigallocatechin (phenolics/flavonoid). Light blue lines represented the inter- and intramolecular H-bonds while orange lines demonstrated the relax H-bond constraints.

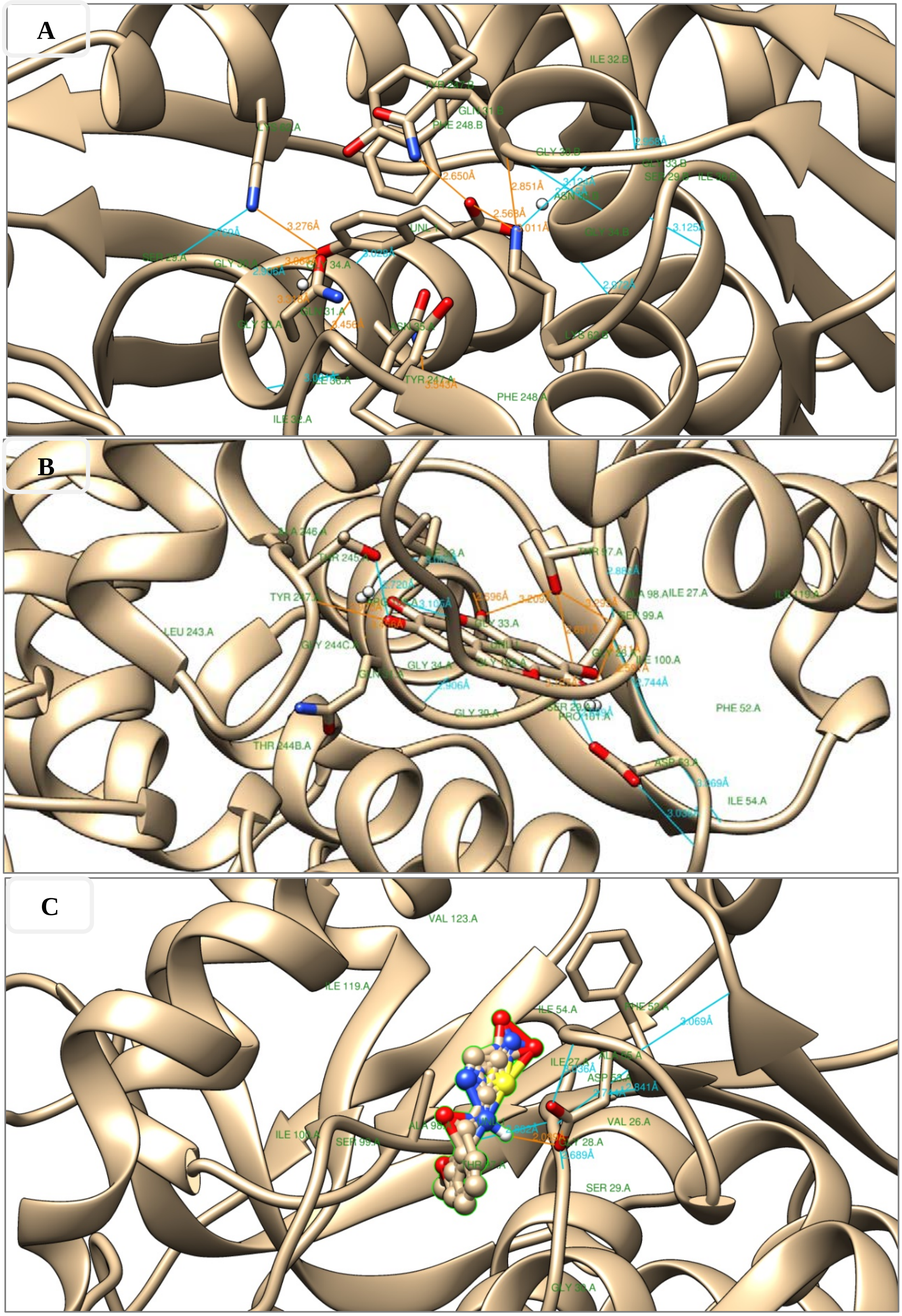
A
ILE 32.B
PHE 248.B
GLY 30.B
2.650Å
2.851Å
3.276Å
2.568Å
3.125Å
SER 29.A
LYS 62.B
TYR 247.A
PHE 248.A
ILE 32.A
B
LEU 243.A
TYR 247.A
GLY 33.A
GLY 244C.A
GLY 34.A
GLY 30.A
ILE 100.A
PHE 52.A
THR 244B.A
ILE 54.A
2.906Å
2.744Å
C
VAL 123.A
ILE 119.A
ILE 54.A
3.069Å
2.841Å
VAL 26.A
SER 29.A
GLY 33.A

Supplementary Figure S2. 3D docked structures toward *Cryptosporidium parvum* lowa II lactate dehydrogenase (LDH) by using UCSF Chimera visualization software. **A.** P-Coumaric acid (phenolics/phenolic acid). **B.** Ellagic acid (phenolics/Ellagic tannins). **C.** Nitazoxanide (NTZ, standard reference drug). Light blue lines represented the inter- and intramolecular H-bonds while orange lines demonstrated the relax H-bond constraints.
